# Spatial detection of mitochondrial DNA and RNA in tissues

**DOI:** 10.3389/fcell.2024.1346778

**Published:** 2024-05-14

**Authors:** Michelle Giarmarco, Jordan Seto, Daniel Brock, Susan Brockerhoff

**Affiliations:** ^1^ Department of Ophthalmology, University of Washington, Seattle, WA, United States; ^2^ Department of Biochemistry, University of Washington, Seattle, WA, United States

**Keywords:** mitochondria, mtDNA, mtRNA, spatial biology, multiplex imaging, zebrafish, RNAscope, photoreceptor

## Abstract

**Background:**

Mitochondrial health has gained attention in a number of diseases, both as an indicator of disease state and as a potential therapeutic target. The quality and amount of mitochondrial DNA (mtDNA) and RNA (mtRNA) can be important indicators of mitochondrial and cell health, but are difficult to measure in complex tissues.

**Methods:**

mtDNA and mtRNA in zebrafish retina samples were fluorescently labeled using RNAscope™ *in situ* hybridization, then mitochondria were stained using immunohistochemistry. Pretreatment with RNase was used for validation. Confocal images were collected and analyzed, and relative amounts of mtDNA and mtRNA were reported. Findings regarding mtDNA were confirmed using qPCR.

**Results:**

Signals from probes detecting mtDNA and mtRNA were localized to mitochondria, and were differentially sensitive to RNase. This labeling strategy allows for quantification of relative mtDNA and mtRNA levels in individual cells. As a demonstration of the method in a complex tissue, single photoreceptors in zebrafish retina were analyzed for mtDNA and mtRNA content. An increase in mtRNA but not mtDNA coincides with proliferation of mitochondria at night in cones. A similar trend was measured in rods.

**Discussion:**

Mitochondrial gene expression is an important component of cell adaptations to disease, stress, or aging. This method enables the study of mtDNA and mtRNA in single cells of an intact, complex tissue. The protocol presented here uses commercially-available tools, and is adaptable to a range of species and tissue types.

## 1 Introduction

Mitochondria, organelles typically associated with energy production, are vital components of nearly all cells. In recent decades new mitochondrial roles have been uncovered, including initiation of cell death (Wang and Youle, 2009), maintenance of calcium (Rizzuto et al., 2012) and redox homeostases (Willems et al., 2015), metabolism of lipids (Houten and Wanders, 2010), and even guiding light in the eye (Ball et al., 2022). They have intricate relationships with the endoplasmic reticulum (Rowland and Voeltz, 2012) that are crucial to the function of neurons and other cells (Wu et al., 2017). Mitochondria are highly dynamic, moving along cytoskeletal networks (Barnhart, 2016), undergoing replication and quality control via fission and fusion (Youle and van der Bliek, 2012; Ni et al., 2015), and sometimes being ejected from cells (Nakajima et al., 2008; Lyamzaev et al., 2022).

Owing to their bacterial symbiont origin, mitochondria possess heterogeneous populations of mitochondrial DNA (mtDNA) that are distinct from genomic DNA (gDNA). The mitochondrial genome is a compact plasmid, with less than 17,000 base pairs encoding 13 critical components of the electron transport chain, plus mitochondrial ribosomal and transfer RNAs needed for translation (Mercer et al., 2011). mtDNA is transcribed into mitochondrial RNA (mtRNA) using a dedicated mtDNA polymerase, and mtRNA is translated into proteins using mitochondrial ribosomes. The remaining >99% of mitochondrial proteins are encoded by gDNA and imported.

Mitochondrial diseases arise from mutations in gDNA that affect mitochondrial proteins, or from mutations to mtDNA itself (Gorman et al., 2016). mtDNA mutations can be difficult to characterize due to the unique maternal inheritance of mitochondria and resulting heteroplasmy (St John et al., 2010). Additionally, certain diseases and aging can lead to aberrant mtDNA levels and mitochondrial dysfunction (Filograna et al., 2021; Sanchez-Contreras and Kennedy, 2022). Despite the importance of mtDNA homeostasis for cells, few tools exist for spatially quantifying mtDNA copy number and/or mtRNA amounts in specific cells of a complex tissue.

We employed *in situ* hybridization (ISH) using RNAscope™ probes (Wang et al., 2012) designed to report either mtDNA, or mtDNA and mtRNA. The strategy is similar to a published chromogenic assay (Chen et al., 2020), but allows for higher-resolution 3-D imaging and simultaneous protein detection using immunohistochemistry (IHC). As a proof of principle, we used this method to explore mtDNA and mtRNA levels in cone and rod photoreceptors during day and night in zebrafish. At night mtRNA but not mtDNA levels increase in both photoreceptor types, which corresponds to increases in mitochondrial number and activity in cones (Giarmarco et al., 2020). This method is adaptable to many species and tissue types; paired with super-resolution imaging and AI-assisted analysis it can quantitatively report mtDNA and mtRNA in individual cells of a complex tissue.

## 2 Materials and equipment

### 2.1 Methods

#### 2.1.1 Animals

Research was authorized by the University of Washington Institutional Animal Care and Use Committee. Wild-type AB *Danio rerio* were raised under standard conditions in the UW SLU Aquatics facility on a 14h/10 h light/dark cycle, with lights on at 9 a.m. Male and female fish were euthanized over an ice bath, followed by cervical dislocation and enucleation. For the 11 p.m. timepoint euthanasia and dissections were performed under infrared light with night vision goggles.

#### 2.1.2 Tissue preparation

For histology, eyes were collected at 9 a.m. and 11 p.m. (4 animals per timepoint), pierced several times across the cornea with a needle, and the vitreous cavity was flushed with 4% paraformaldehyde fixative in phosphate buffered solution (PBS). Following overnight immersion in fixative at 4°C, eyes were rinsed in PBS, then bleached with 10% H_2_O_2_ in PBS overnight at 37°C to clarify the pigmented epithelium. Eyes were cryoprotected in 20% sucrose, the anterior halves dissected away, and eyecups were embedded and frozen in OCT cryomolds. Eyecups were cryosectioned at 20 μm onto slides that contained one section from each timepoint for parallel staining and analysis; slides were stored at −20°C.

For bulk genomic analysis, eyes were collected at 9 a.m. and 11 p.m. (4-5 animals per timepoint), and the retinas were isolated in PBS. Retinas were snap frozen in microfuge tubes over liquid nitrogen and stored at −80°C until DNA extraction.

#### 2.1.3 Probe design

Three commercially-available ACD RNAscope™ probes were used. As a positive control, Dr-polr2a-C3 reported mRNA transcripts from a nuclear-encoded zebrafish housekeeping gene. As a negative control, 3-plex DapB reported transcripts from a bacterial gene. To explore mtDNA and mtRNA we used probes against the mitochondrial gene MT-ND5, which encodes a highly-conserved subunit of respiratory complex I and was identified by the manufacturer as a good candidate reporter. The “coding” probe Dr-MT-ND5 contains sequences antisense to the MT-ND5 gene, binding to regions of the noncoding (sense) mtDNA strand, and to MT-ND5 mtRNA transcripts.

To report mtDNA, we employed a strategy similar to one reported for chromogenic RNAscope™ with paraffin-embedded samples (Chen et al., 2020). The “noncoding” probe Dr-MT-ND5-sense-C3 was custom designed to contain sense sequences of the same mitochondrial gene; it binds regions of the coding (antisense) mtDNA strand. Antisense mtRNA transcripts from the noncoding strand of mtDNA are typically degraded (Pietras et al., 2018; Jedynak-Slyvka et al., 2021), so this probe reports primarily mtDNA.

#### 2.1.4 *In situ* hybridization with RNAscope™

Sections were treated with the RNAscope™ Fluorescent Multiplex v2 kit. The standard protocol for fixed frozen sections was used including pretreatment, probe hybridization and fluorescent labelling, with the following modifications: 1) Target retrieval was reduced to 5 min 2) Protease III digestion was reduced to 15 min 3) Probe and dye concentrations were optimized to best visualize single molecules (see Table 2). 4) Following the final wash, sections were not mounted and instead immediately underwent IHC. Incubations were conducted in staining chambers in a standard incubator. MT-ND5 probes were conjugated to the fluorescent dye Opal™ 520 diluted in RNAscope™ TSA buffer. Polr2a (C3) and DapB (C2) control probes were conjugated to Opal™ 520 and Opal™ 570, respectively.

#### 2.1.5 Enzymatic validation of probes

To verify specificity of mtDNA and mtRNA detection with the MT-ND5 probes, an additional incubation in either PBS or RNase A was performed. Following post-protease water washes and just prior to probe hybridization, sections were incubated 30 min at 37°C in either PBS or 50 μg/mL RNase A in PBS, followed by 3 water washes. This was followed by standard probe hybridization, fluorescent labelling, and IHC. Additional validation strategies are outlined in the Section 3.

#### 2.1.6 Immunohistochemistry

Following RNAscope™, sections underwent IHC using a primary antibody against MTCO1 (a subunit of respiratory complex IV) to report mitochondrial volume. Sections were washed 3 times with PBS, blocked 1 h at room temperature (RT) with blocking buffer, and incubated overnight at RT with anti-MTCO1 diluted in blocking buffer. They were then washed 3 times with PBS, incubated 2 h at RT with Alexa Fluor™ 633-conjugated secondary antibodies and DAPI counterstain, washed 3 times with PBS, and mounted with Vectashield and a coverslip. Table 1 lists primary antibodies used in this study, along with others the authors have found to be compatible for multiplexing IHC with RNAscope™.

**TABLE 1:** Materials and equipment. Primary antibodies suitable for multiplexing IHC with RNAscope^™^ in cryosections.

Tissue species	Structure labeled	Antigen	Supplier	Catalog #	RRID	IHC dilution
Any	Transgenic GFP	GFP	Abcam	ab13970	RRID:AB_300798	1:5,000
Zebrafish	Mitochondria	MTCO1	Abcam	ab14705	RRID:AB_2084810	1:1,000
Zebrafish	Mitochondria	Citrate synthetase	Abcam	ab96600	RRID:AB_10678258	1:500
Macaque	Neuron cytosol	Calbindin	Sigma-Aldrich	C8666	RRID:AB_10013380	1:1,000
Macaque	Synapses	GAD6	Iowa Hybridoma Bank	GAD-6	RRID:AB_528264	1:1,000
Human	Tight junctions	ZO-1	ThermoFisher	40–2,200	RRID:AB_2533456	1:100
Human	Collagen	COLVI	Abcam	ab6588	RRID:AB_305585	1:250
Human	Plasma membrane	CD46	Bio-Rad	MCA2113	RRID:AB_323983	1:100

#### 2.1.7 Imaging and image processing

Imaging was conducted using a Leica SP8 confocal microscope with a ×63 oil objective to capture single Z-stacks and Z-stack montages. LAS-X software was used for acquisition, and images were deconvolved using Leica Lightning in adaptive mode. Images were processed in ImageJ, where mitochondrial clusters of individual rods and short-wavelength cones were identified based on morphology and position in the retina (cyan and yellow boxes in Figure 2A). Single clusters were cropped out for quantification using the MTCO1 mitochondrial channel as a mask. Confocal images presented are maximum intensity projections over 12 µm tissue depth.

#### 2.1.8 Quantification of probe signals

Mitochondrial clusters of rod photoreceptors were found to have well-resolved individual RNAscope™ MT-ND5 puncta. Single cluster Z-stacks (n = 3 cells per condition) were analyzed using the 3D Objects Counter plugin in ImageJ to report the number of puncta per cluster. MT-ND5 puntca in cone clusters were poorly resolved in areas due to very dense packing of mitochondria. Single cluster Z-stacks (n = 30 cells per standard timepoint, n = 12 cells per timepoint for RNase validation) were analyzed using the 3D Objects Counter plugin in ImageJ to instead report total puncta volume relative to overall mitochondrial volume measured using the MTCO1 channel. For both rods and cones, analysis was conducted blind to sample condition and timepoint. Mann-Whitney statistical tests were performed using Microsoft Excel with the Real Statistics resource pack.

#### 2.1.9 DNA extraction and quantification using qPCR

DNA was isolated from frozen retinas (n = 3-4 animals per timepoint, each with three technical replicates) using an AllPrep DNA/RNA Mini Kit, then quality checked via spectrophotometer. mtDNA:gDNA ratios were determined using the qPCR 2^−ΔΔCT^ method (Livak and Schmittgen, 2001) relative to the 9 a.m. timepoint. Mitochondrial-encoded NADH dehydrogenase 1 (*mt-nd1*) served as the mtDNA target and *polg1* was the nuclear-encoded DNA target, a strategy reported previously in zebrafish (Artuso et al., 2012). Primer sequences for these targets are listed in Table 2. qPCR CT values were generated using extracted DNA on the 7500 Fast Real-Time PCR System and SYBR Green Real-Time PCR Master Mix. The number of mtDNA copies per cell was determined according to the following equation (Artuso et al., 2012):

$$mtDNA\;copies=\frac{\left(number\;of\;mtDNA\;copies\right)}{\frac{\left(number\;of\;allele\;of\;nDNA\right)}{2}}$$

**TABLE 2:** Materials and Equipment.

Item	Source	Notes, concentrations
Phosphate buffered solution (PBS)	in-house	0.14 M phosphate buffer in water, pH 7.4
16% paraformaldehyde solution	ThermoFisher, #043368.9M	4% in PBS
30% hydrogen peroxide solution	MilliporeSigma, #HX0640	10% in PBS
20% sucrose solution	in-house	20% w/v sucrose in PBS
Optimal Cutting Temperature (OCT) compound	VWR, #4583	
Cryomolds (10 mm × 10 mm x 5 mm)	VWR, #4565	
SuperFrost Plus Gold slides	ThermoFisher, #15-188-48	
Cryostat	Leica Biosystems, #CM1850	
Hybridization Incubator	Robbins Scientific, #310	
Slide moisture chamber, black	Newcomer Supply, #68432A	
Probe Dr-polr2a-C3	ACD Bio, #559921	1:50 in probe diluent
Probe 3-plex DapB	ACD Bio, #320871	neat
Probe Dr-MT-ND5	ACD Bio, #574591	0.5X in probe diluent
Probe Dr-MT-ND5-sense-C3	ACD Bio, #1204141	1:75 in probe diluent
Probe diluent	ACD Bio, #300041	
Fluorescent Multiplex v2 kit	ACD Bio, #323100	
TSA buffer	ACD Bio, #322809	
Opal™ 520 dye	Akoya Biosciences, #FP1487001KT	1:1,000 for polr2a probe; 1:1,800 for MT-ND5 probes in TSA buffer
Opal™ 570 dye	Akoya Biosciences, #FP1488001KT	1:1,000 for DapB probe in TSA buffer
Monarch RNase A	New England Biolabs, #T3018-2	50 μg/mL (1:400) in PBS
Blocking buffer	in-house	5% normal donkey serum, 1 mg/mL bovine serum albumin, 1% Triton X-100 in PBS
Mouse anti-MTCO1	Abcam, #ab14705; RRID:AB_2084810	1:1,000 in blocking buffer
Goat anti-mouse IgG, Alexa Fluor™ 633 conjugate	ThermoFisher, #A21053	1:500 in blocking buffer
DAPI	Invitrogen, #D1306	1:1,000 in blocking buffer
Vectashield Vibrance	Vector labs, #H1700	
Leica SP8 confocal microscope with LAS-X	Leica Microsystems	
FIJI (ImageJ)	https://fiji.sc/ (Schindelin et al., 2012)	
3D Objects Counter Plugin	https://imagej.net/plugins/3d-objects-counter (Bolte and Cordelieres, 2006)	
AllPrep DNA/RNA Mini Kit	Qiagen, #80204	
NanoDrop™ spectrophotometer	ThermoFisher, #ND-2000C	
Fast Real-Time PCR System	Applied Biosystems, #7500	
DEPC-treated water	ThermoFisher, # R0601	
Zebrafish polg1 primer set	Integrated DNA Technologies	(F) GAG​AGC​GTC​TAT​AAG​GAG​TAC
		(R) GAG​CTC​ATC​AGA​AAC​AGG​ACT
Zebrafish *mt-nd1* primer set	Integrated DNA Technologies	(F) AGC​CTA​CGC​CGT​ACC​AGT​ATT
		(R) GTT​TCA​CGC​CAT​CAG​CTA​CTG
SYBR Green Real-Time PCR Master Mix (2X)	Applied Biosystems, # 4309155	1X in DEPC-treated water
Microscoft Excel with Real Statistics resource pack	Microsoft, Real Statistics	

## 3 Results

### 3.1 Experimental design

The mitochondrial genome consists of a “heavy” coding strand, and a complementary “light” noncoding strand. During transcription mtRNA is generated from both strands; sense transcripts from the coding strand are processed and translated into proteins, transfer RNAs, and ribosomal RNAs (Pietras et al., 2018; Jedynak-Slyvka et al., 2021). mtRNA transcripts from the noncoding strand are typically degraded, except for a few transfer RNAs and one protein (Chomyn et al., 1986; Mercer et al., 2011).

**FIGURE 1:**
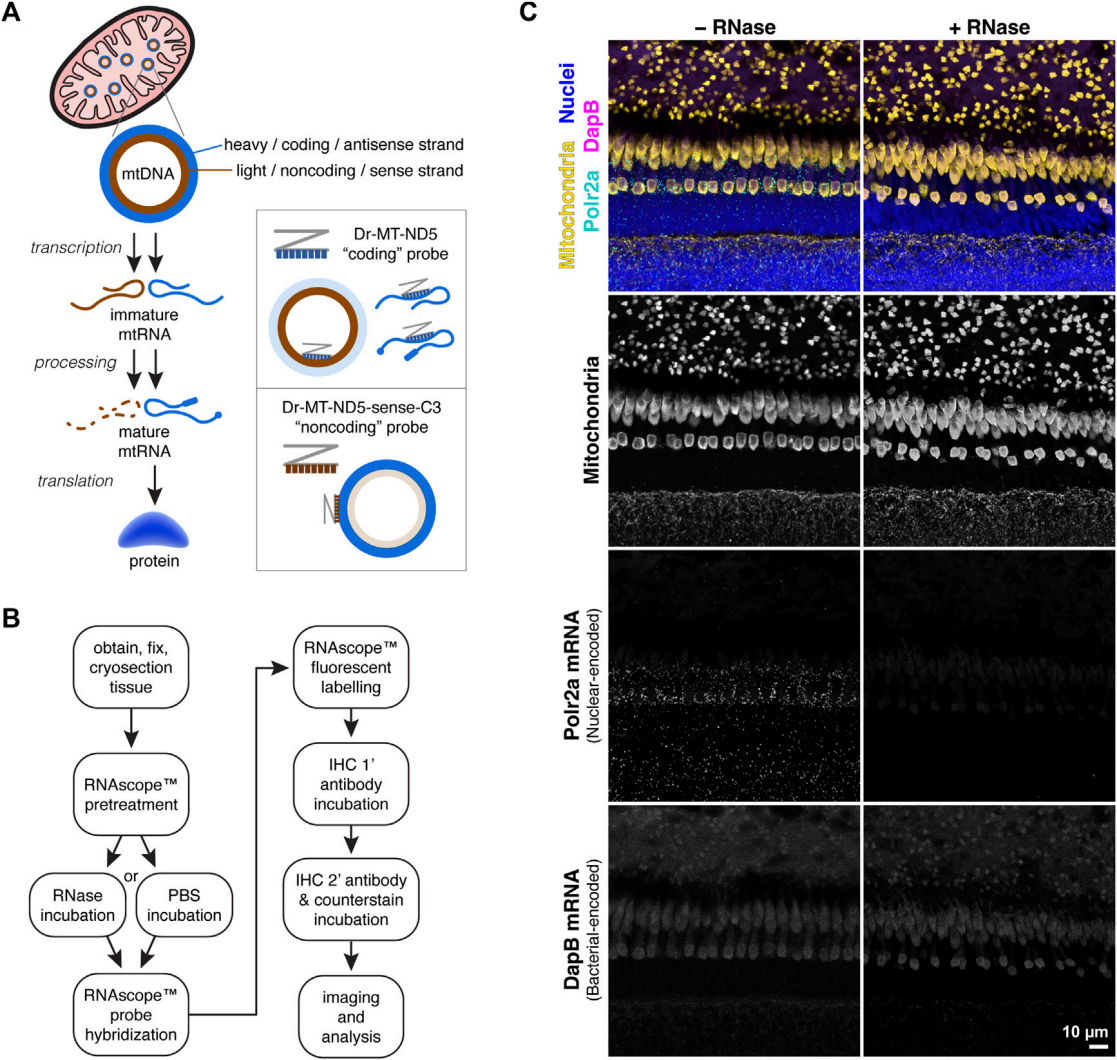
**(A)** Simplified schematic of protein synthesis from mtDNA. mtRNA transcripts from the mtDNA coding strand are processed and translated into protein; transcripts from the noncoding strand are typically degraded. Accordingly, the RNAscope™ Dr-MT-ND5 “coding” probe reports both mtRNA and mtDNA, while the “noncoding” probe reports primarily mtDNA. **(B)** Workflow for mtDNA and mtRNA detection using RNAscope™ and IHC. RNAscope™ steps were carried out using the manufacturer’s protocol for the Fluorescent Multiplex v2 kit, with modifications listed in the methods section. **(C)** Images of zebrafish outer retina stained with a control probe against Polr2a nuclear-encoded mRNA, and a control probe against bacterial-encoded DapB mRNA. RNase treatment eliminates Polr2a mRNA signal.

Figure 1A depicts the strategy for separately labelling mtDNA and mtRNA transcripts using two RNAscope™ probes against each strand of the mitochondrial gene MT-ND5. For consistency in this manuscript, the “coding” MT-ND5 probe sequence is antisense, binding both mtRNA transcripts and the mtDNA light strand. The “noncoding” MT-ND5 probe sequence is sense, binding primarily to the mtDNA heavy strand.

### 3.2 Probe validation strategies

#### 3.2.1 Enzymatic validation

To determine the relative contribution of RNA to probe signals, we employed an optional RNase incubation step prior to probe hybridization (Figure 1B). We used an RNAscope™ positive control probe against messenger RNA (mRNA) transcripts from the nuclear-encoded housekeeping gene Polr2a, and a negative control probe against mRNA transcripts from the bacterial gene DapB. In zebrafish retina sections, the Polr2a probe labeled puncta around nuclei that were absent with RNase pretreatment; no puncta were visible with the DapB probe (Figure 1C).

Further enzymatic validation using DNase to digest mtDNA has been reported for paraffin sections (Chen et al., 2020). We attempted this repeatedly with fixed frozen retina sections but results were difficult to interpret, as DNases removed most of the RNA signal from the positive control mRNA probe. One explanation is that structural damage to the tissue from DNase is more severe in fixed frozen sections, causing RNA to float away before probe hybridization. This is an important control that could be successful in other tissues, but likely necessitates paraffin sections.

#### 3.2.2 ISH and IHC multiplexing

To confirm that MT-ND5 probe signals were localized to mitochondria, we expanded the RNAscope™ procedure to include subsequent immunolabelling (Figure 1B). The pretreatment conditions required for RNAscope™ destroyed antigenicity of some primary antibody targets, while antigenicity was unaffected or improved for others. Table 1 lists primary antibodies we have used to successfully immunolabel various cell structures following ISH in zebrafish, macaque, or human tissue cryosections.

**FIGURE 2:**
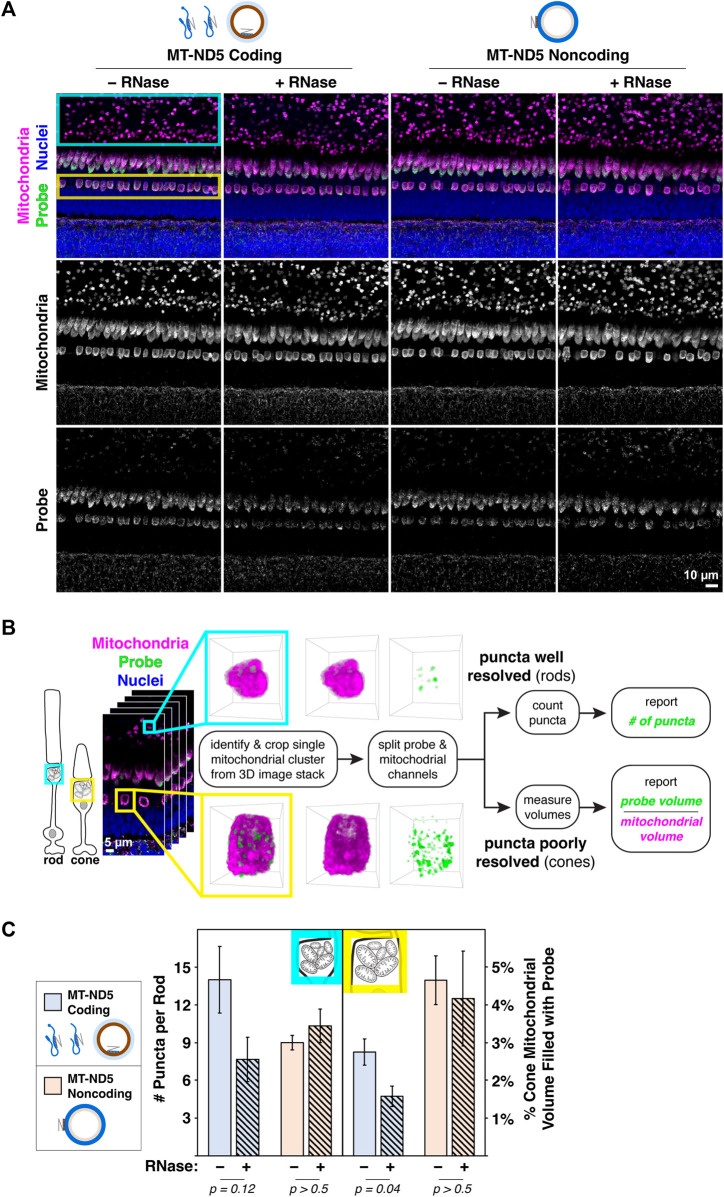
**(A)** Images of zebrafish outer retina showing signals from RNAscope™ MT-ND5 coding and noncoding probes. Cyan and yellow boxes indicate respective locations of rod and cone mitochondrial clusters used for quantification. RNase was used to determine signal contributions of RNA for each probe. All sections were counterstained for nuclei and mitochondria via IHC. Green–probe (ISH), magenta–mitochondria (IHC), blue–nuclei. **(B)** Workflow for quantification of MT-ND5 probe signals as either puncta counts or percent of mitochondrial volume. **(C)** Quantification of signals from coding and noncoding probes, including RNase conditions. Error bars represent standard error of the mean; *p* values determined using a Mann-Whitney test.

A primary antibody against MTCO1, a subunit of respiratory complex IV, displayed a robust mitochondrial staining pattern (Figure 1C, second row) following ISH that is typical of zebrafish retina stained using conventional IHC (Giarmarco et al., 2020). When ISH against MT-ND5 and IHC against MTCO1 were multiplexed, signals from coding and noncoding probes were confined to mitochondria (Figure 2A), demonstrating that the MT-ND5 probes are not binding off-target nuclear-encoded genes. Mitochondria in zebrafish photoreceptors are tightly packed and do not immunolabel uniformly (see U-shaped structures in Figure 1C, second row), but IHC is still useful for broad morphometrics like cluster size and outline.

Immunolabelling with antibodies against dsRNA or dsDNA is also useful for validation of mitochondrial probes. We did not employ this method in our samples because IHC quantification is confounded by limitations with antibody penetration. However, dual labeling with RNAscope™ and anti-dsRNA has been reported for cultured primate cells (Liu et al., 2018).

#### 3.2.3 Bulk analysis

**FIGURE 3:**
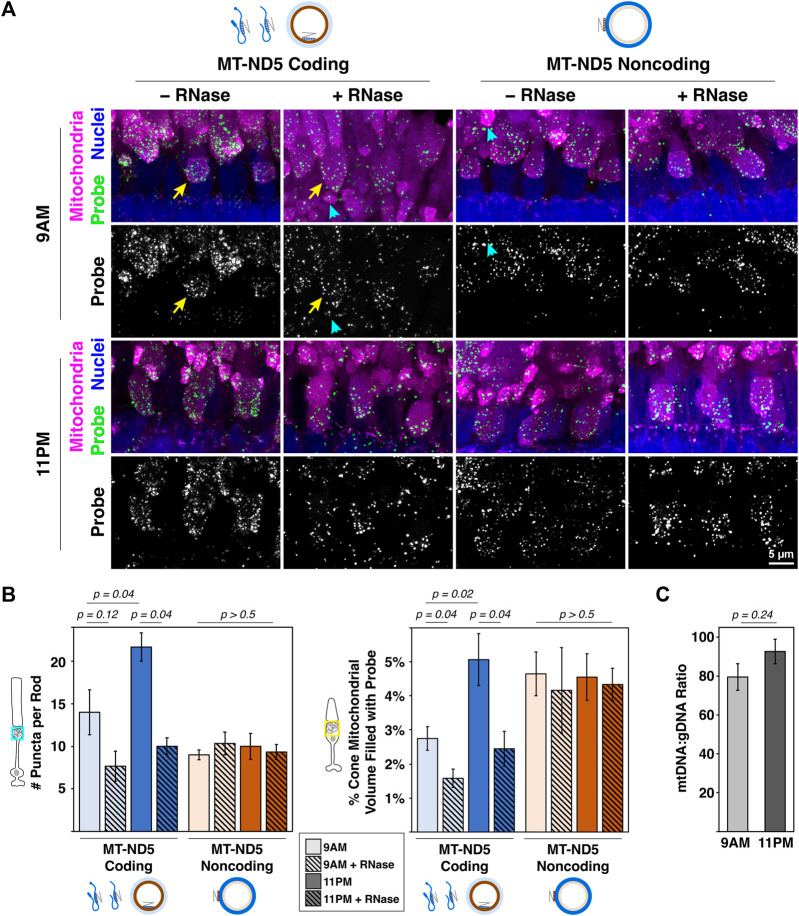
**(A)** Sample images of zebrafish photoreceptor mitochondrial clusters from 9 a.m. to 11 p.m. labeled with RNAscope™ MT-ND5 coding or noncoding probes. RNase was used to determine signal contributions of RNA for each probe. All sections were counterstained for nuclei and mitochondria via IHC. Cyan arrowheads and yellow arrows indicate examples of single rod and cone mitochondrial clusters used for quantification, respectively. Green–probe (ISH), magenta–mitochondria (IHC), blue–nuclei. **(B)** Quantification of signals from coding and noncoding probes at 9 a.m. and 11 p.m., including RNase conditions. **(C)** Ratios of mtDNA to gDNA measured using RT-qPCR from whole zebrafish retinas at 9 a.m. and 11 p.m.. For all graphs, error bars represent standard error of the mean; *p* values determined using a Mann-Whitney test.

Given the difficulty using DNase as a control for RNAscope TM superscript on cryosections, bulk analysis using quantitative PCR (qPCR) can be used to confirm findings about mtDNA. By comparing the number of mitochondrial and nuclear genomes in a sample, mtDNA copy number per cell can be calculated (Artuso et al., 2012). While cell-specific information is lost, patterns in mtDNA copy numbers found using RNAscope TM superscript should be consistent with observations made using qPCR. We demonstrate validation using qPCR in a proof-of-principle experiment below (Figure 3C).

### 3.3 Quantification of mtDNA and mtRNA

As a demonstration of the multiplex labeling strategy, we sought to quantify mitochondrial RNAscope™ signals in photoreceptors of zebrafish retina, where mitochondria are concentrated in a dense cluster. We employed two strategies for quantification, outlined in Figure 2B.

For rods, quantification of MT-ND5 signals was straightforward. Individual puncta within a cluster were well resolved, and we used the 3D Object Counter plugin for ImageJ to report the number of puncta per cluster. For cones, quantification was limited by the dense packing of cone mitochondria, and the resolution of our microscope. Despite careful titration of MT-ND5 probes and their conjugated dyes, we could not reliably resolve individual puncta in all areas of a cluster. Instead, we used the 3D Objects Counter plugin for ImageJ to report probe volume as a percentage of overall cluster volume determined from IHC.

RNase pretreatment modestly reduced signal from the coding probe but not the noncoding probe (Figure 2C). Insensitivity of the noncoding probe to RNase confirms that it does not appreciably bind mtRNA.

### 3.4 Demonstration quantifying mtDNA and mtRNA in photoreceptors

Mitochondrial dynamics in rod photoreceptors are not well characterized, but cone clusters undergo daily remodeling. Their mitochondria are smaller and more numerous at night (Giarmarco et al., 2020) when energy demands are highest (Okawa et al., 2008), and it is not known how mtDNA and mtRNA change. In retina samples collected during the day (9 a.m.) and at night (11 p.m.), both MT-ND5 probes yielded robust signal in rod and cone mitochondrial clusters (Figure 3A). A subset of these samples was pretreated with RNase to determine the relative contribution of mtRNA to MT-ND5 signals.

Figure 3B summarizes the analyses of MT-ND5 coding and noncoding probe signals. Only the coding probe was significantly sensitive to RNase pretreatment (solid v. hashed bars), confirmation that the noncoding probe primarily reports mtDNA. In rods, the number of coding probe puncta increased 35% between 9 a.m. and 11 p.m., and noncoding puncta were not significantly different. A similar pattern was observed in cones, where volume of the coding probe increased 45% between 9 a.m. and 11 p.m., but noncoding probe volumes were unchanged.

Given the increase in mitochondrial number previously reported at night, we were surprised to find no change in mtDNA levels using the MT-ND5 noncoding probe. We further validated this finding by performing qPCR to report relative mtDNA copy number in whole retinal homogenates collected at 9 a.m. and 11 p.m.. Ratios of mtDNA:gDNA determined via qPCR were not significantly different between 9 a.m. and 11 p.m. (Figure 3C), consistent with observations made using RNAscope™.

Together this suggests that a burst of mitochondrial transcription, evidenced by increases in coding probe signal, could support a larger population of mitochondria at night in cones. Overall mtDNA levels remain unchanged, indicating that either existing mitochondrial genomes are simply divided among the growing population, and/or that some amount of mtDNA turnover is occurring. Alternately, it is possible that mtDNA levels rise at a timepoint that was not measured in this study.

## 4 Discussion

We present a method for spatial quantification of mtDNA and mtRNA in fixed frozen tissue sections. It employs a dual-probe strategy for RNAscope™ ISH similar to a published method in paraffin sections (Chen et al., 2020), coupled with IHC for unambiguous labeling of mitochondria. The protocol uses commercially-available reagents, can be carried out over 2–3 days, and is broadly adaptable to many species and tissues. It will enable researchers to characterize mitochondrial gene expression in single cells, and understand the mitochondrial adaptations that occur during disease, stress and aging.

Conventional methods for measuring mtDNA and mtRNA such as RT-qPCR often require tissue homogenization, which presents challenges to studying specific cells. In the example of the retina, photoreceptors are just part of a tissue comprised of around 100 cell types, making it difficult to discern their contribution to changes in mitochondrial gene expression above the retinal milieu. Recent advances in single-cell RNA sequencing have enabled the study of mtDNA in retinal cell subpopulations (Liu et al., 2022), but some neuron types are not represented. Further, tissue digestion and cell sorting conditions can induce stress-related changes to gene expression (Denisenko et al., 2020).

This method has several advantages over chromogenic ISH and bulk nucleotide analyses. In addition to preserving cells morphologically in their native microenvironment, rapid fixation of tissue for histology reduces risk of stress-related gene expression changes that occur during tissue dissociation. Multiplexing ISH with IHC enables counterstaining of mitochondria or other cell-identifying markers, making for unambiguous quantification of ISH signals. We found several primary antibodies suitable for ISH and IHC multiplexing (Table 1). Lastly, the high resolution afforded by confocal microscopy allows for visualization of single mtDNA or mtRNA molecules in 3-D, and subsequent quantification.

RNAscope™ is a versatile platform amenable to many species and tissue types, and the design of double Z probe sets enables accurate target labelling with virtually no off-target binding (Wang et al., 2012). We present a simple RNAscope™ application, but depending on the kit and sample, it can be used to visualize up to 48 targets. Its application in the study of mtDNA and mtRNA is somewhat recent but gaining popularity (Blumental-Perry et al., 2020; Lee et al., 2023; Sriram et al., 2024).

Following careful biostatistics-aided probe selection, additional validation steps are critical. Antibodies detecting mitochondrial proteins and dsDNA are useful to this end, and probes targeting several mitochondrial genes would also help validate results. In the case of the zebrafish MT-ND5 probes, RNase pretreatment confirmed that the coding probe reports mtDNA and mtRNA, while the noncoding probe reports primarily mtDNA. We attempted pretreatment with DNase as additional enzymatic validation, but it was not compatible with RNAscope™ on fixed frozen sections. We relied on qPCR comparing total amounts of gDNA and mtDNA as confirmation of our results.

In an example analysis of mtDNA and mtRNA in zebrafish photoreceptors, mtRNA levels increased 35%–45% between 9 a.m. and 11 p.m., a finding consistent with cone mitochondrial proliferation (Giarmarco et al., 2020) and increased photoreceptor energy consumption at night (Okawa et al., 2008). Interestingly mtDNA levels remained stable, highlighting the complicated nature of mitochondrial gene expression. It is possible that daily turnover of mitochondrial genomes keeps mtDNA levels stable, as cycles of mtRNA expression and degradation support changes to the mitochondrial population. This supports the idea that increases in cone mitochondrial number are driven primarily by increased gene expression, rather than a net increase in mtDNA copy number.

While our example analysis uncovered significant differences in mtRNA levels, there are limitations to quantification depending on the cell type and mitochondrial organization. In zebrafish rods, mitochondria are large and reside in a small cluster. mtDNA and mtRNA molecules were clearly resolved within rod clusters, making quantification straightforward. In contrast, cones have hundreds of smaller mitochondria concentrated in a dense cluster, and mtDNA and mtRNA molecules are very crowded. In such cases, it is important to titrate the concentrations of probe and fluorescent dye to best achieve individual puncta; even then we were unable to resolve individual puncta across the entire cone cluster. Reporting volume fractions was an effective strategy for demonstration of the method, however counts of individual puncta are most accurate. This could be achieved by utilizing super-resolution imaging (Liu and Rask-Andersen, 2022), and/or deploying machine learning analysis tools (Burkert et al., 2023).

Probe selection is an important consideration when using RNAscope™ to report mtDNA and mtRNA. We selected the MT-ND5 gene based on success with the commercially available MT-ND5 coding probe for zebrafish, and had the MT-ND5 noncoding probe custom designed by the manufacturer and their bioinformatics team. Because both probes target the same gene, they likely contain complementary sequences that would bind each other in solution when attempting to duplex both probes. Indeed, our attempts at duplexing coding and noncoding probes were difficult to interpret, despite tandem hybridization steps and labelling with spectrally separate dyes. To successfully duplex coding and noncoding probes on the same section, two different mitochondrial genes would need to be targeted.

Another factor to keep in mind when interpreting results from this method is probe affinity. In our example of zebrafish cones, 3-D electron microscopy in a previous study found ∼180 mitochondria per cluster at 9 a.m. In a few instances where RNAscope™ mtDNA puncta in a cluster were resolved well enough for counting using 3-D segmentation and manual counting, we found roughly half that number of mtDNA molecules. While it is possible that some mitochondria in this system lack a genome, another explanation is that the probe did not bind every mitochondrial genome. Coding and noncoding probes may not have the same affinity for their target sequences, so it is important to include enzymatic validation, use several probes, and exercise caution when making direct numerical comparisons.

In conclusion, we have optimized a method of multiplexed ISH and IHC that can detect mtDNA and mtRNA at single molecule resolution. Using machine learning and super resolution imaging strategies, this method holds promise for precisely quantifying determinants of mitochondrial health, such as mtDNA copy number and gene expression. This spatial information can address new questions about mitochondrial biology at the level of an individual mitochondrion, in different locations within a cell or tissue, or in particular stages of the mitochondrial life cycle.

## Data Availability

The original contributions presented in the study are included in the article/Supplementary Material, further inquiries can be directed to the corresponding author.
